# Some Modern Aspects of Diabetes. II

**Published:** 1893-10-14

**Authors:** 


### Some Modern Aspects of Diabetes.—II.

It will be remembered that in our last article we showed that if the whole pancreas is excised in certain animals diabetes results; if a part only is taken out, diabetes does not follow. We propose now to point out other advances that have lately been made in our knowledge of this disease.

Until recently it was stated that salicylic acid, amyl nitrite, carbonic oxide, urari, turpentine, chloral, and some other less important substances, could cause sugar to appear in the urine of healthy persons taking them. The foundation for this statement was that they cause the urine to reduce the copper in Fehling's solution. We now know that none of these bodies induce glycosuria, for the urine of those who have taken them, even if it does reduce Fehling's solution, will not give the fermentation test for sugar. What the urine really contains in these cases is glycuronic acid, which will reduce blue copper solutions. Phloridzin and phloretin, however, when taken by the mouth lead to the appearance in the urine of genuine dextrose, and consequently it was thought by some that we had in these two substances agents capable of artificially producing diabetes. Minkowski has, however, shown that this is not so, for in the phloridzin glycosuria there is no increase of sugar in the blood, and phloridzin will cause glycosuria in animals in whom excision of the pancreas does not lead to diabetes. It appears that phloridzin really exerts a local action on the kidneys, and that the sugar is produced in these organs.

Minkowski next turned his attention to the fate of the various carbohydrates, if they were given to animals in whom the pancreas had been excised, and who were, consequently, suffering from diabetes. He found that if small amounts of laevulose were given, very little of this carbohydrate appeared in the urine, from which he fairly concluded that the greater part was used in the body. Large quantities of laevulose caused, however, a considerable increase in the elimination of dextrose in the urine, while some of the laevulose passed out unchanged in this fluid. Inulin is the starch which bears the same relation to laevulose as ordinary starch does to dextrose, it is met with in large quantities in several plants, notably dahlia tubers. Now it was found that, strange to say, the ingestion of inulin leads to the passage of dextrose and not laevulose in the urine, although we should have expected that laevulose would appear, seeing that the administration of ordinary starch leads to the passage of dextrose. The practical outcome of these results is that it is improbable that either laevulose or inulin will be found to be of much use in the treatment of human diabetes. Minkowski also showed that the administration of cane sugar, or of milk sugar, causes dextrose to appear in the urine of animals in whom the pancreas has been excised, thus confirming the clinical experience of the dieting of patients suffering from diabetes. Finally, the last result he obtained from excision of the pancreas was that after the operation the glycogen disappeared from the liver in a few days.

It is well known that an enormous literature has grown up concerning the cause of diabetic coma, and as our knowledge stands at present it appears that the coma bears some relation to—if, indeed, it is not caused by—the appearance of oxybutyric acid in the urine, for Stadelmann states that it only occurs in the urine of those diabetics who suffer from coma, and this accords with Minkowski's observation that it was sometimes present—and more particularly shortly before death—in the urine of animals whose pancreas had been excised. This view, that oxybutyric acid is the cause of the coma, explains the fact that acetone and acetic acid can be found in the urine of diabetics, and, indeed, coma has been attributed to each of these, for acetic acid is a product of oxidation of $\beta$ oxybutyric acid, and acetic acid easily breaks up into acetone and carbonic acid. The further expansion of this view is that the oxybutyric acid, and probably other fatty acids, act as acids because of the withdrawal of ammonia from the blood. Indeed, it is stated that a sufficiently accurate way of estimating the amount of these acids in the urine is to make a quantitive analysis of the ammonia; the more there is of this the less oxybutyric acid. It is very interesting to note that Vaughan Harley, working from the standpoint of injecting large amounts of grape-sugar into the blood of animals, and so producing coma, also concludes that the right treatment for diabetic coma is to administer alkalies, and he agrees with Stadelmann's recommendation to inject a solution of sodium carbonate subcutaneously or intravenously. Roque, Devic, and Hugeouneuq, have also independently shown that a diminution of the alkalinity of the blood is an important factor in the production of diabetic coma. It is only fair, however, to add that Minkowski looks upon the presence of oxybutyric acid in the urine as a complication of diabetes and not as the cause of the coma, for he urges that many of his animals did not show it, and it is met with in other diseases.

Another direction in which our knowledge of diabetes has increased is in the discovery that sometimes peripheral neuritis is present as a complication. It is, however, rare, but when met with pain is nearly always a prominent symptom, and often it is remarkably severe. Defects of motion are far less common, but if the patient suffers from any paralytic symptoms the legs are nearly sure to be weaker than the arms. Ataxic symptoms are very rare. It is a remarkable fact that whilst peripheral neuritis is such an uncommon complication of diabetes, half the patients, as Judson Bury has shown, have lost their knee jerk, and if cases in old patients are excluded, the proportion is much higher than a half. It is difficult to know to what this can be due to, if not to peripheral neuritis, for in the cases examined the spinal cord has been found to be healthy, but no explanation is forthcoming for the fact that other symptoms of neuritis than the abolition of the knee jerk are not met with more often. This discovery of peripheral neuritis in diabetes derives additional interest from the fact that for a long while it has been known that diabetic optic neuritis is sometimes seen, and it serves to explain isolated observations formerly made from time to time, in which some nerves were found to be diseased, although then this was used to explain the diabetes on the view that it was due to vaso-motor dilatation in the liver. A good instance of such neuritis are the cases of neuritis of the splanchnics in diabetes described by Hale White in vol. 36 of the "Pathological Transactions."

The last point in the recent advances of our knowledge of diabetes to which we would direct attention is the improvement which has been made in the dietary for this disorder. Dr. Saundly, in the *Birmingham Medical Review*, has recently pointed out the value of Clark's starchless biscuits, and he also gives excellent receipts for making almond cakes, cocoanut cakes, and Iceland moss pudding, all of which contain but little saccharine matter. The biscuits are the most important of these foods, for the other articles are liable to produce indigestion. Soya biscuits made from soya beans are a great addition to our list of diabetic foods, for they contain very little starch or sugar, and many patients like them much better than the gluten bread; and, at anyrate, they are excellent as an occasional relief from the monotony of it. The soya beans contain much oil, and are largely used as an article of food in China and Japan. Soya sauce is made from them, and they make an excellent soup for diabetics. Some cases showing how useful they are have been recently recorded in *The Practitioner* by Hale White. Aleuronat, another new food for diabetics, is a gluten prepared from wheat, and it has been strongly recommended by Ebstein. It is a dry yellow powder, which is said to contain only 7 per cent. of carbohydrates. It is disagreeably gritty, and so difficult to make into anything palatable that it cannot be largely used.

